# The life course process of coming out on the rural Northern Great Plains

**DOI:** 10.1093/geroni/igaf122.3670

**Published:** 2025-12-31

**Authors:** Bryce Van Vleet, Heather Fuller, Alyssa Willis

**Affiliations:** Eastern Mennonite University, Harrisonburg, Virginia, United States; North Dakota State University, Fargo, North Dakota, United States; North Dakota State University, Fargo, North Dakota, United States

## Abstract

Identity development for queer individuals involves recognizing non-majority sexual or gendered feelings. Emerging research has identified milestones in queer identity development, indicating coming out can be a developmental process across adulthood. Yet, these studies have excluded rural-dwelling adults who have unique geographical identities. Thus, the current project sought to examine the developmental process of coming out as queer in rural America from retrospective recollections of midlife and older adults (M Age = 61.5). Oral history data was collected beginning in 2017 from regional queer trailblazers in the Northern Great Plains, 28 of whom grew up in a rural area. Abductive thematic analysis was conducted using Elder’s life course theory as the guiding deductive framework and resulted in three themes: antecedents to coming out, coming out processes, and consequences of coming out. Participants were likely to feel pressured by traditional gender norms and thus out of place in their rural community of origin. Participants also noted the importance of representational media or people in their rural community that helped them come to terms with their identity. Many participants also indicated using therapy and/or migrating to an urban area to accept their identity. Social networks also helped support or challenge them during their identity exploration and achievement. Findings provide important geographic nuance to the developmental literature and suggest that the availability of cultural resources in rural areas profoundly impacts the ease of queer identity development among rural-dwelling queer people.

